# A Single Visualization Technique for Displaying Multiple Metabolite–Phenotype Associations

**DOI:** 10.3390/metabo9070128

**Published:** 2019-07-02

**Authors:** Mir Henglin, Teemu Niiranen, Jeramie D. Watrous, Kim A. Lagerborg, Joseph Antonelli, Brian L. Claggett, Emmanuella J. Demosthenes, Beatrice von Jeinsen, Olga Demler, Ramachandran S. Vasan, Martin G. Larson, Mohit Jain, Susan Cheng

**Affiliations:** 1 Cardiovascular Division, Department of Medicine, Brigham and Women’s Hospital, Harvard Medical School, Boston, MA 02115, USA; 2 National Institute for Health and Welfare, FI 00271 Helsinki, Finland; 3 Department of Medicine, Turku University Hospital and University of Turku, FI 20521 Turku, Finland; 4 Departments of Medicine & Pharmacology, University of California San Diego, La Jolla, CA 92093, USA; 5 Department of Statistics, University of Florida, Gainesville, FL 32611, USA; 6 Framingham Heart Study, Framingham, MA 01701, USA; 7 Preventive Medicine, Department of Medicine, Brigham and Women’s Hospital, Boston, MA 02115, USA; 8 Preventive Medicine, Department of Medicine, Boston University Medical Center, Boston, MA 02215, USA; 9 Biostatistics Department, School of Public Health, Boston University, Boston, MA 02215, USA; 10 Smidt Heart Institute, Cedars-Sinai Medical Center, Los Angeles, CA 90048, USA

**Keywords:** metabolomics, visualizations, clinical outcomes research, epidemiology

## Abstract

To assist with management and interpretation of human metabolomics data, which are rapidly increasing in quantity and complexity, we need better visualization tools. Using a dataset of several hundred metabolite measures profiled in a cohort of ~1500 individuals sampled from a population-based community study, we performed association analyses with eight demographic and clinical traits and outcomes. We compared frequently used existing graphical approaches with a novel ‘rain plot’ approach to display the results of these analyses. The ‘rain plot’ combines features of a raindrop plot and a conventional heatmap to convey results of multiple association analyses. A rain plot can simultaneously indicate effect size, directionality, and statistical significance of associations between metabolites and several traits. This approach enables visual comparison features of all metabolites examined with a given trait. The rain plot extends prior approaches and offers complementary information for data interpretation. Additional work is needed in data visualizations for metabolomics to assist investigators in the process of understanding and convey large-scale analysis results effectively, feasibly, and practically.

## 1. Introduction

Advances in metabolomics technologies have enabled the generation of large-scale metabolomics measures in human studies [1]. Accordingly, newer generation visualization tools are needed to assist with the analyses and interpretation of these increasingly high-dimensional and complex data sets. Several resources now offer a variety of techniques for visualizing metabolomics data structure and exploring the inter-relations between individual and groups of metabolites [2,3,4,5,6,7,8,9,10,11,12,13,14,15,16,17,18,19,20,21]. 

The most commonly used methods for visually analyzing and representing associations between metabolites and outcomes are borrowed from conventional statistics and other biological fields [2]. Methods such as Manhattan plots, bar and scatter plots, and heatmaps are commonly used for visualizing information on the association between metabolites and outcomes. However, creating visualizations that can facilitate the interpretation of multi-level analyses, including information regarding associations among multiple metabolites and multiple outcomes, continues to pose a special challenge. We have therefore developed a visualization technique that expands upon existing approaches to enable the display of results from multiple simultaneous analyses relating metabolites and clinical phenotypes.

## 2. Methods

For development of these visualization methods, we used a metabolomics dataset comprising >500 bioactive lipids assayed by high resolution liquid chromatography-mass spectrometry (LC-MS) in a subset N = 1447 participants (age 66 ± 9 years, 54% women) of the Framingham Heart Study offspring cohort. LC-MS was performed according to previously described protocols [22]. In brief, plasma samples were prepared and analyzed using a Thermo Vanquish UPLC coupled to a high resolution Thermo QExactive orbitrap mass spectrometer. Metabolites were isolated from plasma using protein precipitation with organic solvent followed by solid phase extraction. Extracted metabolites were underwent chromatographic separation using reverse phase chromatography whereby samples were loaded onto a Phenomenex Kinetex C18 (1.7 µm, 2.1 × 100 mm) column and eluted using a 7 minute linear gradient starting with water:acetonitrile:acetic acid (70:30:0.1) and ending with acetonitrile:isopropanol:acetic acid (50:50:0.02). LC was coupled to a high resolution Orbitrap mass analyzer with electrospray ionization operating in negative ion mode, with full scan data acquisition across a mass range of 225 to 650 m/z. Thermo .raw data files were converted to 32-bit centroid .mzXML using Msconvert (Proteowizard software suite), and resulting .mzXML files were analyzed using Mzmine 2.21, as described [22]. For the present analyses, 16% of analytes had >10% missing values; we replaced missing values for metabolites with 0.25 x the minimum observed value for that metabolite, as reported previously [23]. Metabolite variables were then natural logarithmically transformed and standardized to facilitate cross-metabolite comparisons.

**Figure 1:**
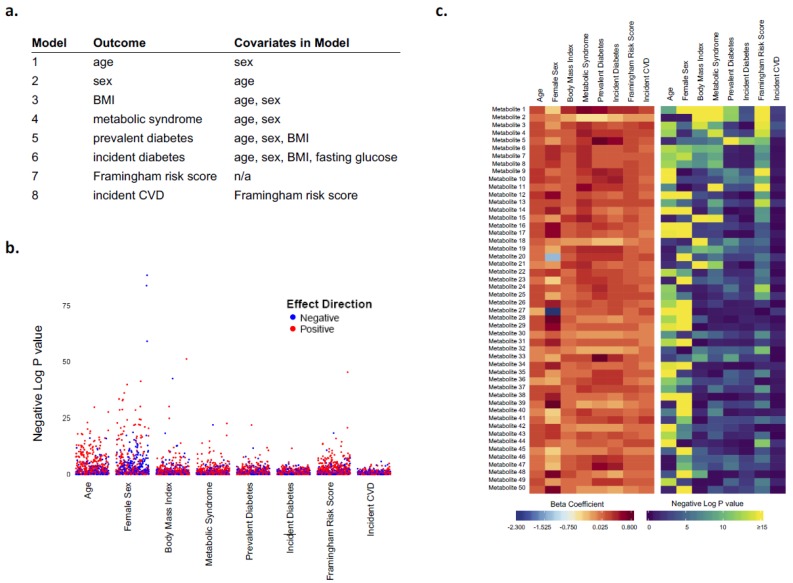
Visualization of complex metabolomics data. For a set of statistical models (**a**) performed in a large human study, for example, a Manhattan plot (**b**) can display the degree to which a wide panel of metabolites is associated with different outcomes although the magnitude of these associations is not conveyed. Pairing of heatmaps can display magnitude as well as directionality and significance for each metabolite association (**c**), although between-metabolite comparisons of associations across all outcomes is not easily discernible.

Given the biological importance of lipid metabolites with respect to cardiometabolic disease traits, we performed multivariable regression analyses to examine the relation of each metabolite with several clinical traits and outcomes, in the following order: age, female sex, body mass index, metabolic syndrome [24], prevalent diabetes [25], incident diabetes [25], Framingham Risk Score [26] as a measure of prevalent cardiovascular risk assessed at examination cycle 8, and incident hard cardiovascular disease [27] (Figure 1). We displayed the results of these relational analyses using a variety of techniques, including Manhattan plots (for one outcome at a time, with results ordered by mass-to-charge [m/z] value), bar and scatter plots (for one outcome at a time, with bars representing magnitude and directionality of estimates, and scatter dots representing P values), heatmaps (p’s or beta’s only), rain plot (beta’s, p’s, trends across a panel). We created heatmaps and rainplots with metabolites both unclustered and clustered based on hierarchical clustering. Details regarding the coding schema used to develop the rainplot approach and select specific parameters for the type of data displayed, for a given set of outcomes, are provided at:  https://github.com/biodatacore/2017.09-rainplots. Detailed instructions and the R code for recreating the graphics in this paper is also provided at the site. All analyses and data visualizations were performed using R v3.4.1. 

## 3. Results

All approaches to visualizing the results of association analyses demonstrated a range in the degree to which different metabolites were related to the different outcomes of interest. The extent and type of information conveyed varied across the visualization techniques, as summarized in Table 1 and detailed below.

Manhattan plots display P values for each model run and highlight statistically significant associations of all metabolites with each outcome across all the metabolites analyzed and ordered from left to right along the x axis by mass-to-charge ratio (*m/z*) (Figure 1 and Figure S1). The marked significance of metabolite associations with sex as well as BMI, compared with the other outcomes, is more clearly demonstrated when the results for all outcomes are displayed in a faceted plot with a shared y axis. Although colored dots can provide information regarding the directionality of associations, along with corresponding P value, the magnitude of each association is not easily conveyed. This issue can be addressed using parallel heatmaps, a commonly applied visualization approach for high-dimensional ‘omics’ data, wherein one plot depicts magnitude of effect and directionality (e.g., beta coefficients) while the other depicts corresponding statistical significance (e.g., P value) (Figure 1 and Figure S2). Although such an approach allows for global visualization of data, discerning clear patterns within or between metabolites can be difficult, especially across multiple heatmap plots and with increasing data set size. In turn, the bar and scatter plots display both the directionality and magnitude of associations, along with P values, for each outcome (Figure S3). The extent to which the same metabolite is positively or negatively associated with different outcomes is not as easily discernible. However, the overall trend of generally more positive or more negative associations observed between a given outcome (e.g., age and Framingham Risk Score) and a large panel of metabolites is clearly displayed. Exceptions to such trends are also highlighted.

**Figure 2:**
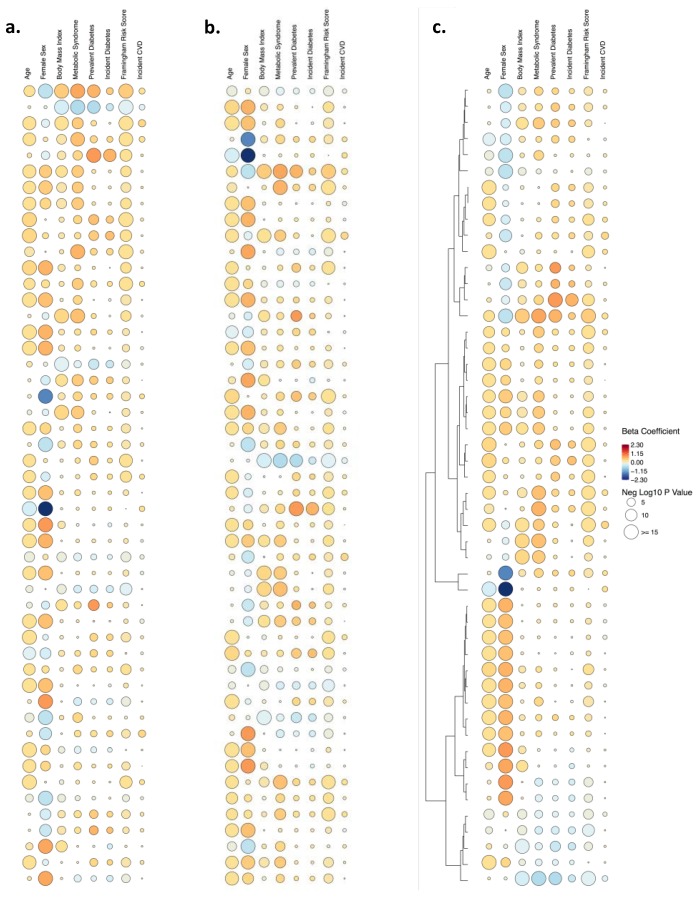
Rain plots. Results are ordered top to bottom by smallest to largest P value (**a**), mass-to-charge ratio (**b**), and by clustering (**c**).

For studies that involve multiple staged experiments or statistical models, several options exist for visualizing analysis results. Our objective was to overcome limitations associated with discerning clear patterns within or between metabolites, especially across multiple plots and outcomes, and with increasing data set size. We therefore combined the visualization concepts offered by the conventional heatmap and previously reported raindrop plot methods; [28] the latter should not be mistaken for another similarly named method used to visualize collections of likelihoods and distributions [29]. As seen in the adapted ‘rain plot’ shown in Figure 2, directionality and magnitude of estimates for the top 50 metabolites associated with the selected outcomes are displayed using a color fill scale and the corresponding significance level is represented by size of the circle (i.e., rain ‘droplet’). The metabolites can also be ordered top to bottom by smallest to largest P value, mass-to-charge ratio, and by clustering.

## 4. Discussion

A ‘rain plot’ approach combines the information from paired heatmaps into a single plot that emphasizes two types of information: (i) between-outcome comparisons, such as the extent to which most metabolites in this panel are associated with certain outcomes (e.g., age, sex) more than others; and, (ii) between-metabolite comparisons, such as the extent to which certain metabolites are associated with an aggregate measure of clinical cardiovascular disease risk (e.g., Framingham risk score) with or without concurrent relations to major component risk factors (e.g., diabetes risk for Metabolites 3 and 4, compared to for Metabolites 1 and 2). Between-metabolite comparisons, in particular, can facilitate identification of potentially important biological differences underlying the observed results of relating multiple metabolites to multiple phenotypes. 

The rain plot visualization emphasizes two types of comparisons: (1) between-outcome results, and (2) between-metabolite results. For between-outcome comparisons, visually scanning for vertical patterns of large sized or deeply colored droplets (i.e., droplet ‘streams’) serves to highlight those outcomes that are the most broadly associated with a given panel of metabolites. As shown in Figure 2, this particular panel of bioactive lipids appears more globally associated with older age, sex, and Framingham Risk Score. A special feature of the rain plot is its emphasis on potentially important between-metabolite comparisons. For instance, certain metabolites (i.e., 20 and 27) are very strongly associated with sex (Figure 1 and Figure 2). For each of these metabolite rows, a visual scan from left to right clarifies the relatively lower degrees of association for these metabolites with other outcomes of interest. The plot also visually clarifies interesting findings between the top-most prioritized metabolites. For instance, Metabolite 1 is positively associated with older age, male sex, and greater metabolic as well as cardiovascular disease risk. Conversely, Metabolite 2 is associated with lower metabolic risk, but is not significantly associated with either age or sex (Figure 2). The plot also highlights a finding for Metabolite 3 that distinguishes this analyte from Metabolites 1 and 2: while similarly associated with both greater Framingham Risk Score and risk for incident cardiovascular disease events, Metabolite 3 is not associated with prevalent or incident diabetes (Figure 2). In effect, Metabolite 3 appears associated with both prevalent and future cardiovascular risk through a biological pathway that is likely distinct from diabetes risk. Similar between-metabolite comparisons are possible across the entire plot. 

As analytical chemistry methods continue to mature, resulting in larger and more complex metabolomics data, there is a growing need for ways to visually understand and interpret the relations of these high-dimensional data with multiple outcomes of interest. Using a dataset of metabolite measures performed in a population scale cohort, we compared several existing techniques that are commonly used for visualizing associations between metabolites and clinical outcomes. To improve these prior methods, we developed and demonstrate the potential utility of a rain plot approach to maximally render the multiple types of information that can be derived from the observed relationships between a panel of metabolites and a set of clinical traits and outcomes. We anticipate that this approach may be further extended and applied to alternate study designs using different types of molecular phenotyping data—as part of the ongoing effort to effectively, efficiently, and feasibly convey the results of large-scale, high-dimensional data analyses [30,31].

## Figures and Tables

**Table 1:** Dimension of information offered by different visualization methods.

	Visualization Method			
Dimension of Information	Manhattan Plot	Bar and Scatter Plots	Heatmap	Rain Plot
Example	Figure 1 and Figure S1	Figure S3	Figure 1 and Figure S2	Figure 2
Significance of associations with an outcome	X	X		X
Magnitude of associations with an outcome		X	X	X
Directionality of associations with an outcome	X	X	X	X
Clustering			X	X
Significance of associations with multiple outcomes	X		X	X
Magnitude of associations with multiple outcomes				X
Directionality of associations with multiple outcomes				X

## Supplementary Materials

The following are available online at https://www.mdpi.com/2218-1989/9/7/128/s1, Figure S1: Manhattan plots; Figure S2: Parallel heat maps; Figure S3: Bar and scatter plots.
