# Supplementary material for: A Single Visualization Technique for Displaying Multiple Metabolite–Phenotype Associations
Source: Metabolites. 2019 Jul 2;9(7):128. doi: 10.3390/metabo9070128 (PMC6680673; doi:10.3390/metabo9070128)
Supplement: Supplementary file 1 [file metabolites-09-00128-s001.pdf]

Supplementary Materials

Plasma samples were prepared and analyzed using a Thermo Vanquish UPLC coupled to a high resolution Thermo QExactive orbitrap mass spectrometer. Metabolites were isolated from plasma using protein precipitation with organic solvent followed by solid phase extraction. Extracted metabolites were underwent chromatographic separation using reverse phase chromatography whereby samples were loaded onto a Phenomenex Kinetex C18 (1.7um, 2.1x100mm) column and eluted using a 7 minute linear gradient starting with water : acetonitrile : acetic acid (70:30:0.1) and ending with acetonitrile : isopropanol : acetic acid (50:50:0.02). LC was coupled to a high resolution Orbitrap mass analyzer with electrospray ionization operating in negative ion mode, with full scan data acquisition across a mass range of 225 to 650 m/z. Thermo .raw data files were converted to 32-bit centroid .mzXML using Msconvert (Proteowizard software suite), and resulting .mzXML files were analyzed using Mzmine 2.21, as described.[17] For the present analyses, 16% of analytes had >10% missing values; we replaced missing values for metabolites with 0.25 x the minimum observed value for that metabolite, as reported previously.[20] Metabolite variables were then natural logarithmically transformed and standardized to facilitate cross-metabolite comparisons.

Given the biological importance of lipid metabolites with respect to cardiometabolic disease traits, we performed multivariable regression analyses to examine the relation of each metabolite with several clinical traits and outcomes, in the following order: age, female sex, body mass index, metabolic syndrome,[21] prevalent diabetes,[22] incident diabetes,[22] Framingham Risk Score[23] as a measure of prevalent cardiovascular risk assessed at examination cycle 8, and incident hard cardiovascular disease[24] (Figure 1). We displayed the results of these relational analyses using a variety of techniques, including Manhattan plots (for one outcome at a time, with results ordered by mass-to-charge [m/z] value), bar and scatter plots (for one outcome at a time, with bars representing magnitude and directionality of estimates, and scatter dots representing P values), heatmaps (p's or beta's only), rain plot (beta's, p's, trends across a panel). We created heatmaps and rainplots with metabolites both unclustered and clustered based on hierarchical clustering. Details regarding the coding schema used to develop the rainplot approach and select specific parameters for the type of data displayed, for a given set of outcomes, are provided at: https://github.com/biodatacore/2017.09-rainplots. All analyses and data visualizations were performed using R v3.4.1.

Supplementary Figures

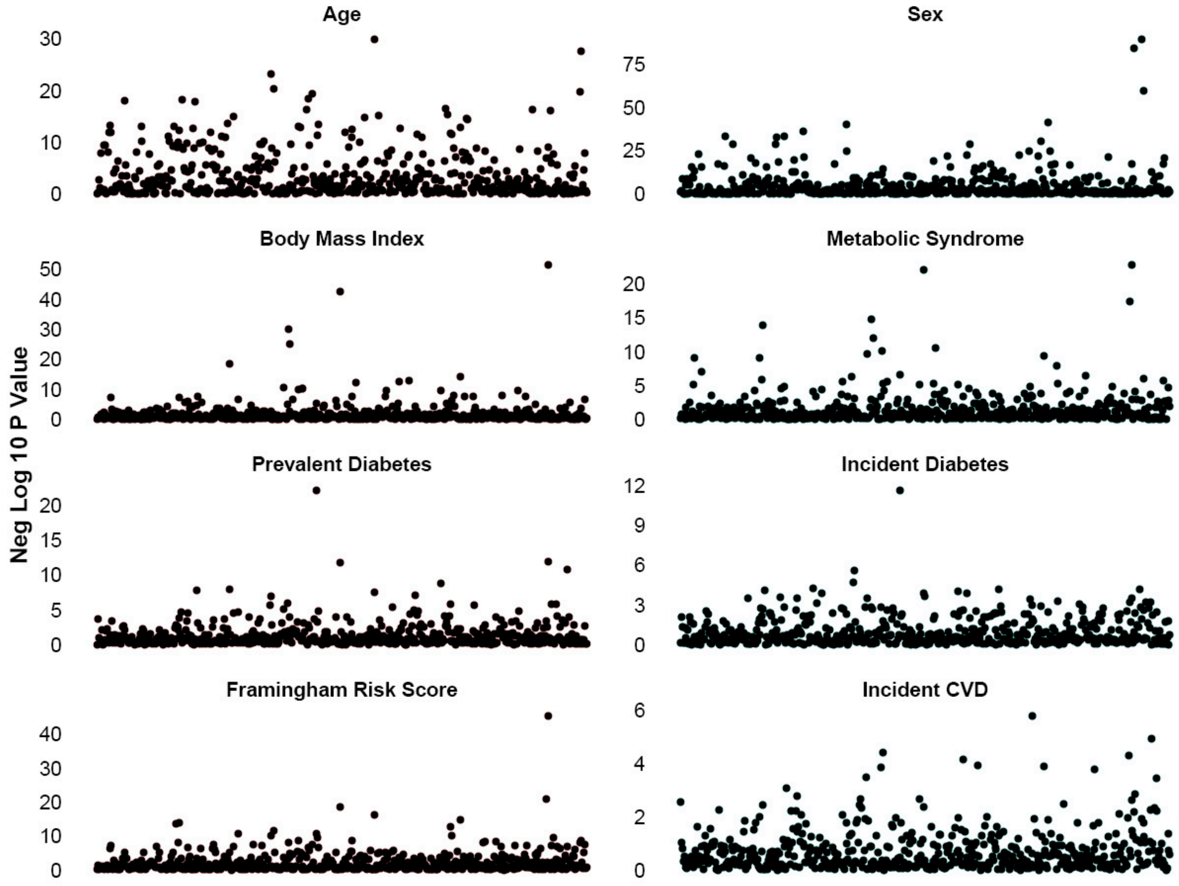

(**a**)

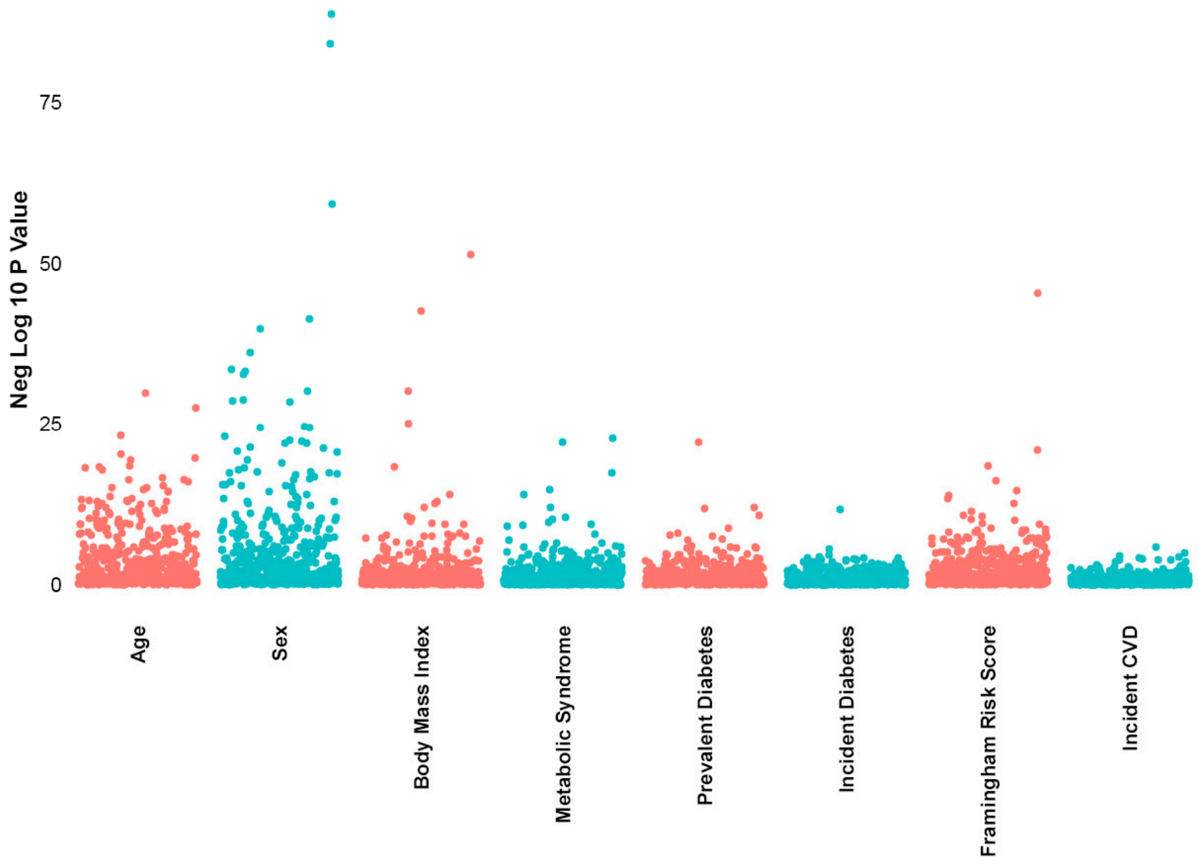

(b)

Figure S1. (a) Panel a. (b) Panel b. Manhattan plots. A panel shows Manhattan plots displaying the distribution of P values for associations between metabolites (ordered by mass-to-charge ratio along the x-axis) and each clinical phenotype separately. Panel a shows Manhattan plots for all clinical traits separately. Panel b shows Manhattan plots for all clinical traits combined into a single visualization with a single y-axis displaying a common maximal value to facilitate comparisons between results across outcomes.

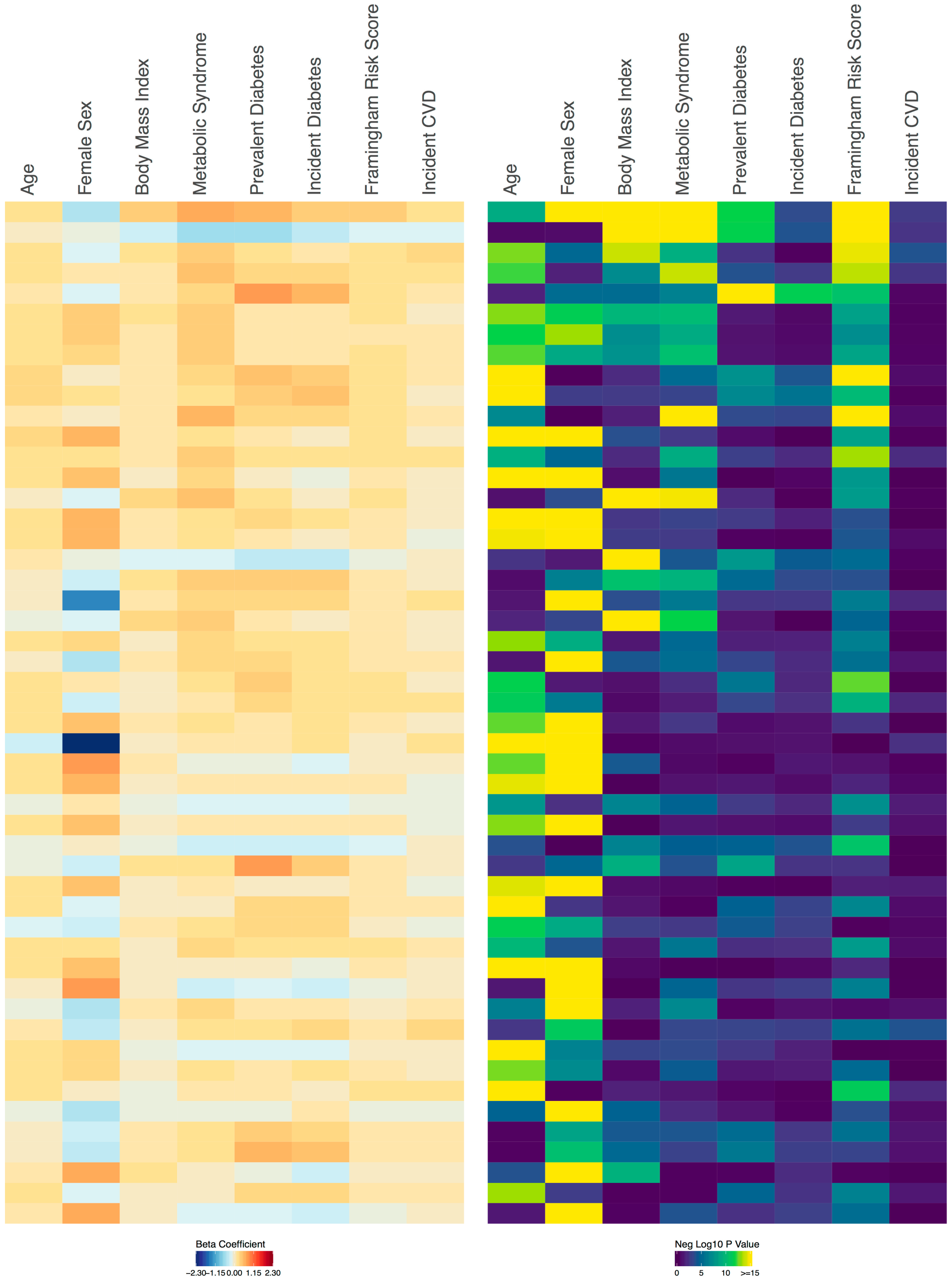

Age
Female Sex
Body Mass Index
Metabolic Syndrome
Prevalent Diabetes
Incident Diabetes
Framingham Risk Score
Incident CVD
Beta Coefficient
−2.30 −1.15 0.00 1.15 2.30
Neg Log10 P Value
0 5 10 >=15

(a)

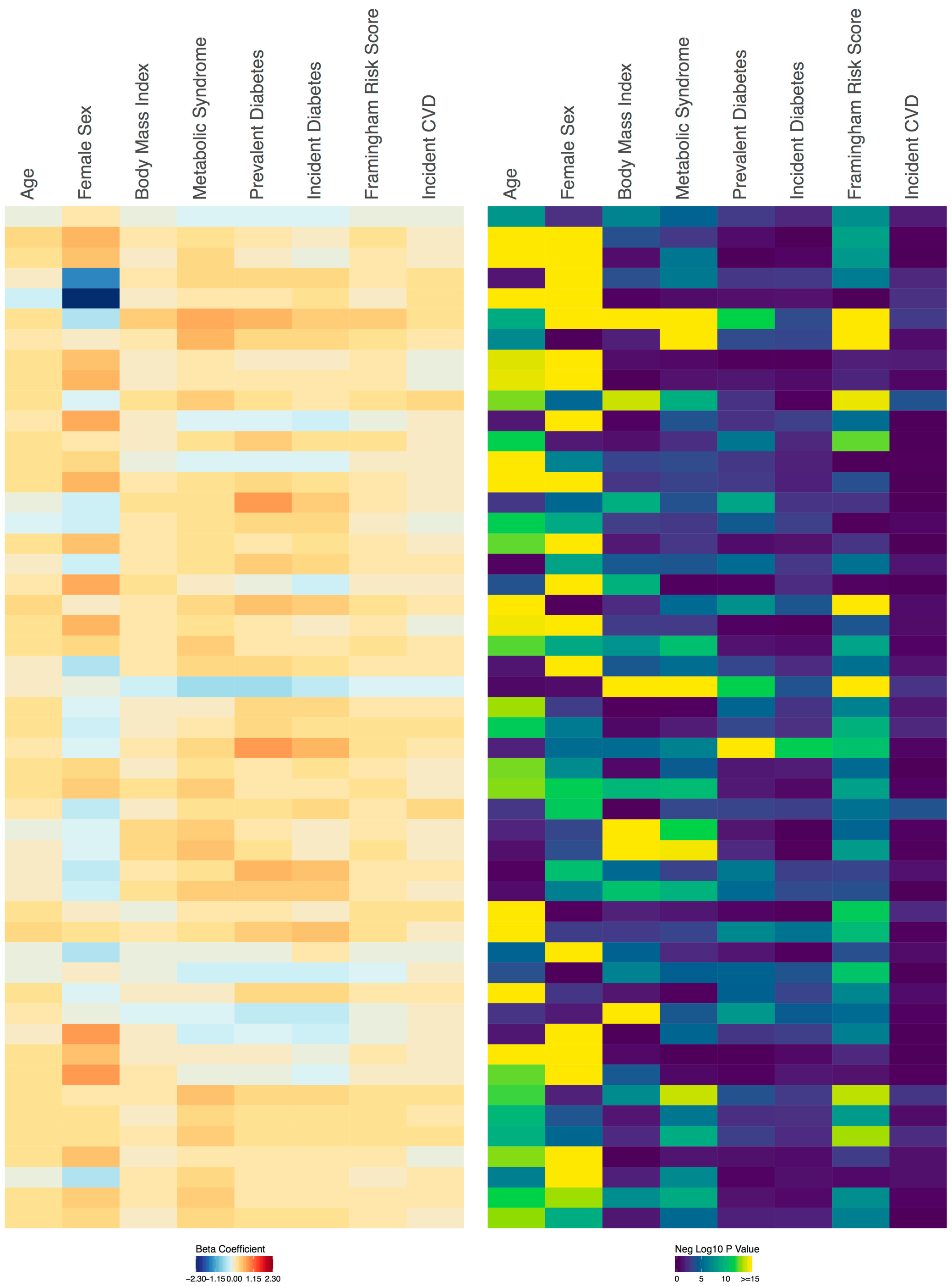
Age
Female Sex
Body Mass Index
Metabolic Syndrome
Prevalent Diabetes
Incident Diabetes
Framingham Risk Score
Incident CVD
Age
Female Sex
Body Mass Index
Metabolic Syndrome
Prevalent Diabetes
Incident Diabetes
Framingham Risk Score
Incident CVD
Beta Coefficient
−2.30 −1.15 0.00 1.15 2.30
Neg Log10 P Value
0 5 10 >=15

(b)

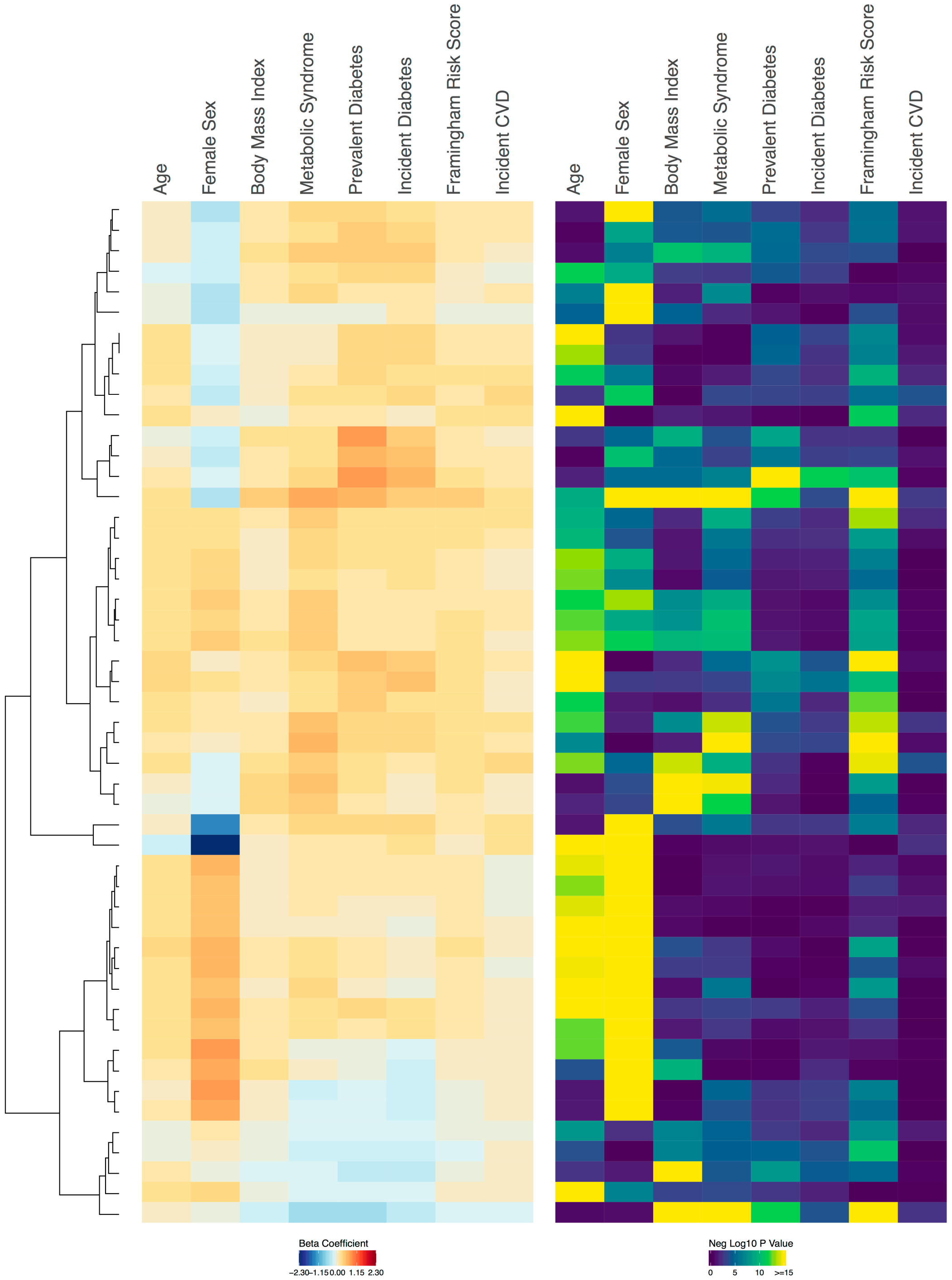

(c)

Figure S2. (a) Panel a. (b) Panel b. (c) Panel c. Parallel heat maps. Parallel heat maps display beta coefficients (left plot) and corresponding P values (right plot) for the top 50 metabolites associated with the clinical traits and outcomes listed as column headings. Results are ordered top to bottom by smallest to largest P value (Panel a), mass-to-charge ratio (Panel b), and by clustering (Panel c).

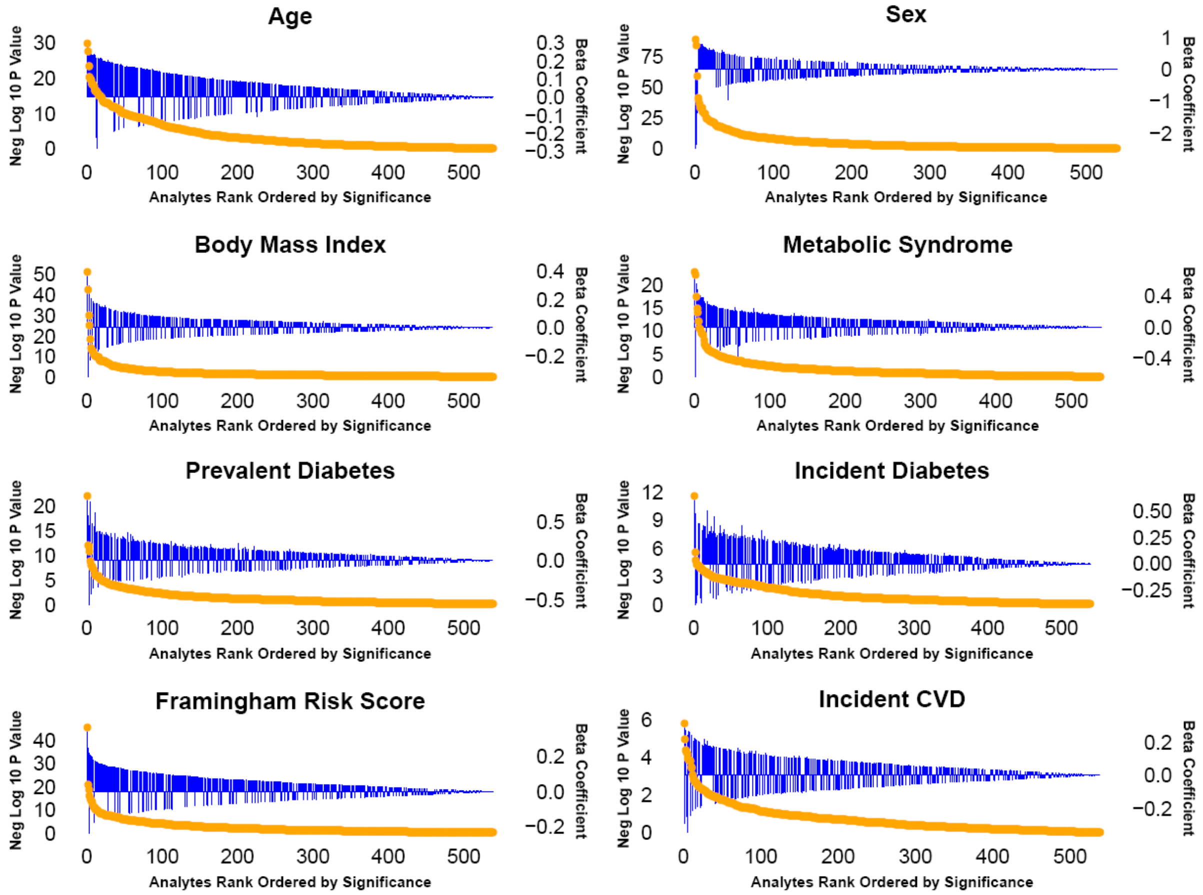
Age
Sex
Body Mass Index
Metabolic Syndrome
Prevalent Diabetes
Incident Diabetes
Framingham Risk Score
Incident CVD
Neg Log 10 P Value
Beta Coefficient
Analytes Rank Ordered by Significance

(a)

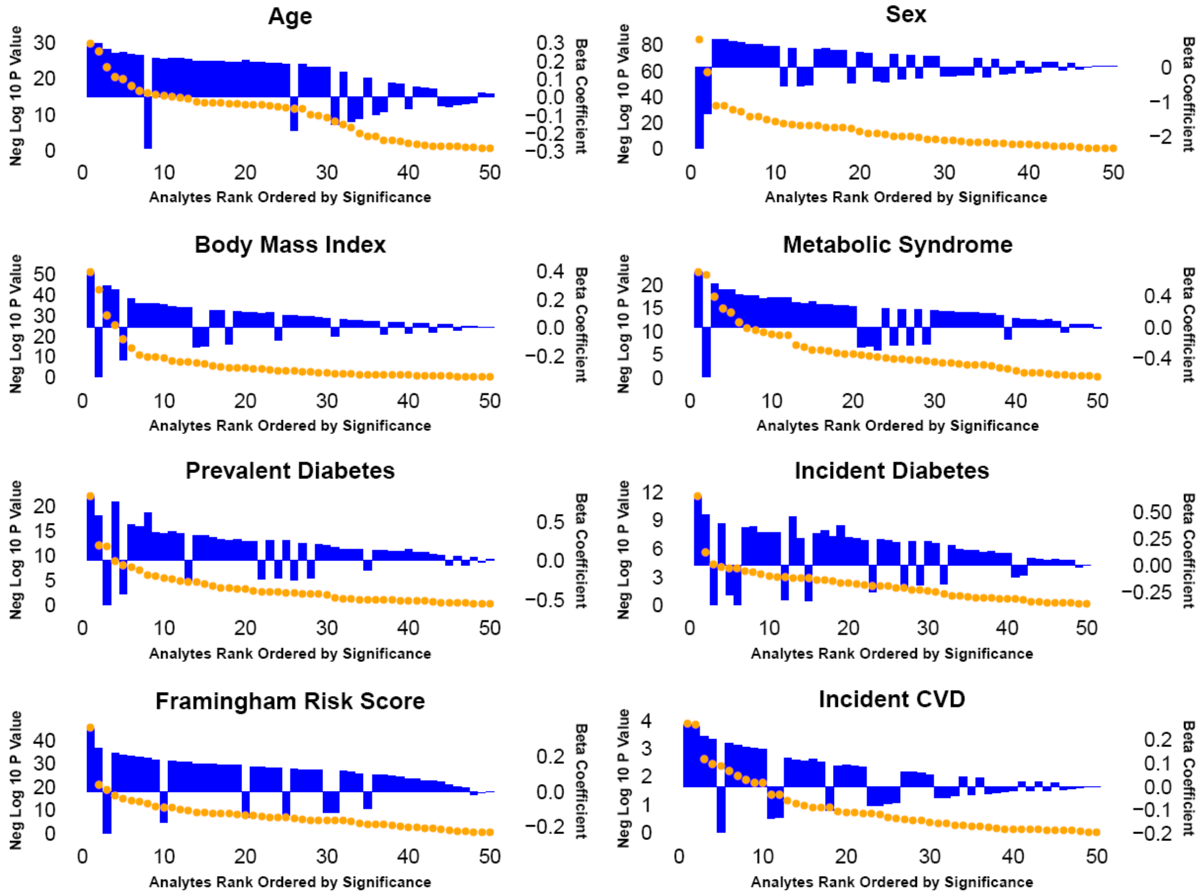

(b)

Figure S3. (a) Panel a. (b) Panel b. Bar and scatter plots. Bar and scatter plots display both the beta coefficients and corresponding P values for associations between metabolites and different clinical traits or outcomes (one per plot) with the order of metabolites ordered left to right from smallest to largest P value. Panel a displays results for all analyses, and Panel b displays results for the top 50 associated metabolites.
